# Engineered Repressible Lethality for Controlling the Pink Bollworm, a Lepidopteran Pest of Cotton

**DOI:** 10.1371/journal.pone.0050922

**Published:** 2012-12-04

**Authors:** Neil I. Morrison, Gregory S. Simmons, Guoliang Fu, Sinead O’Connell, Adam S. Walker, Tarig Dafa’alla, Michelle Walters, John Claus, Guolei Tang, Li Jin, Thea Marubbi, Matthew J. Epton, Claire L. Harris, Robert T. Staten, Ernest Miller, Thomas A. Miller, Luke Alphey

**Affiliations:** 1 Oxitec Limited, Oxford, United Kingdom; 2 Department of Zoology, University of Oxford, Oxford, United Kingdom; 3 Animal Plant Health and Inspection Service, Plant Protection and Quarantine, Centers for Plant Health Science and Technology, United States Department of Agriculture, Phoenix, Arizona, United States of America; 4 Animal Plant Health and Inspection Service, Plant Protection and Quarantine, Centers for Plant Health Science and Technology, United States Department of Agriculture, Salinas, California, United States of America; 5 Department of Entomology, University of California Riverside, Riverside, California, United States of America; U. Kentucky, United States of America

## Abstract

The sterile insect technique (SIT) is an environmentally friendly method of pest control in which insects are mass-produced, irradiated and released to mate with wild counterparts. SIT has been used to control major pest insects including the pink bollworm (*Pectinophora gossypiella* Saunders), a global pest of cotton. Transgenic technology has the potential to overcome disadvantages associated with the SIT, such as the damaging effects of radiation on released insects. A method called RIDL (Release of Insects carrying a Dominant Lethal) is designed to circumvent the need to irradiate insects before release. Premature death of insects’ progeny can be engineered to provide an equivalent to sterilisation. Moreover, this trait can be suppressed by the provision of a dietary antidote. In the pink bollworm, we generated transformed strains using different DNA constructs, which showed moderate-to-100% engineered mortality. In permissive conditions, this effect was largely suppressed. Survival data on cotton in field cages indicated that field conditions increase the lethal effect. One strain, called OX3402C, showed highly penetrant and highly repressible lethality, and was tested on host plants where its larvae caused minimal damage before death. These results highlight a potentially valuable insecticide-free tool against pink bollworm, and indicate its potential for development in other lepidopteran pests.

## Introduction

The sterile insect technique (SIT) is a pesticide-free method of controlling pest insects. It works through the mass-production and release of large numbers of radiation-sterilised insects [1], [2]. The sterile males mate with wild females and, if enough are released over a sufficient period, the wild pest population reduces in size. As a species-specific control method, SIT offers considerable environmental benefits and a chemical-free option where agricultural producers are increasingly restricted by limitations to permitted chemical residues on crops.

For SIT, mass-produced insects are sterilised by ionising radiation prior to release. Sterility results from radiation-induced chromosomal damage in developing sperm and ovaries, causing dominant lethal mutations. The chromosomes of Lepidoptera are, however, more resistant to damage by irradiation than those of other insects [3], due to their diffuse (or holokinetic) centromere. They therefore require a higher dose of radiation for sterility to be induced. For example, SIT programmes for two moth pests - the cotton pest, pink bollworm (*Pectinophora gossypiella* Saunders), and the fruit pest, codling moth (*Cydia pomonella* L.) - typically use gamma radiation doses of 200 Gy and 150 Gy, respectively [4], whereas Mediterranean fruit fly (medfly, *Ceratitis capitata* Wiedemann) programmes use 80–90 Gy [5]. Moreover, even these doses in moths confer full sterility in females but not in males [4], [6], and are used as a compromise to minimise the radiation dose. One problem associated with the relatively high doses of radiation required to sterilise Lepidoptera is potential somatic damage and reduced competitiveness in treated insects [3], [7]. Such doses may also impair sperm transfer to the female spermathecae [8], potentially causing the mated female to seek further mates. It is likely that a full, sterilising dose of radiation not only induces sterility, but impairs the ability of the released insects to reduce mating between native counterparts [9]–[13].

Potential negative effects of irradiation can be avoided by treating the insects with a reduced sub-sterilising radiation dose, which in Lepidoptera can confer sterility in the F_1_ progeny of irradiated adults. This effect, called F_1_ sterility or inherited sterility, has been proposed as a means of avoiding the negative effects of full-dose irradiation on moth performance in the field [14]. F_1_ sterility was successfully used in eradicating an outbreak of the painted apple moth, *Teia anartoides* (Walker), in New Zealand [15]. However, regular bi-sex releases of partially sterile moths could lead to the presence in the field of unmarked sterile F_1_ larvae and adults, potentially increasing crop damage and confusing monitoring efforts. For field monitoring of SIT control programmes, and especially for SIT eradication campaigns, presence of F_1_ larvae and adults can be problematic [16]. In practice, F_1_ sterility programmes are difficult to implement despite the benefits of using a reduced radiation dose.

A genetic variant of SIT - the “release of insects with a dominant lethal” (RIDL) [17] - can provide the effect of sterility without the need for irradiation, and has been developed in medfly [18] and the dengue vector mosquito *Aedes aegypti* (L.) [19]. These insects carry a dominant lethal gene, repressible by tetracycline (or suitable analogues, such as chlortetracycline) supplied during their larval feeding stage. After release into the field, progeny die in the absence of the dietary additive. As with ‘sterilisation’ by irradiation, this is not agametic sterilisation, rather gametes are produced but dominant lethal mutations – radiation-induced or engineered – cause mortality in progeny [20], [21]. We have also developed RIDL strains of fruit flies and mosquitoes in which the lethal phenotype is female-specific, termed female-specific RIDL (fsRIDL) [22]–[24]; in contrast to bi-sex RIDL, in which the lethal phenotype is expressed in both sexes. This may be essential where adult females are damaging, and may also provide some efficiency improvements [25], [26] and additional benefits in managing resistance to other interventions in an integrated pest management (IPM) context [27], [28]. However it also requires a system for sex-specific gene expression. Furthermore, the benefits of male-only release have not been as unequivocally established for moths as for various Diptera.

We sought to develop bi-sex RIDL strains of pink bollworm. This moth is a major global pest of cotton, and a subject of an on-going SIT programme in south-western USA and Northern Mexico. An engineered strain expressing a fluorescent protein marker was previously developed with the aim of improving reliability of monitoring released moths and was successfully tested in the field [29], [30]. A RIDL strain, marked in the same way, would offer cotton growers a method of SIT-type pink bollworm control without the need to irradiate the moths and negate the impact of moth escapes from the rearing facility. Divergence between Lepidoptera and Diptera occurred at least 190 million years ago [31]. Considering such evolutionary distance, the function of a similar RIDL system in a moth - after its demonstration in medfly and *Ae. aegypti* - was uncertain.

Stable, germ-line transformation of pink bollworm has previously been achieved using the transposable element, *piggyBac* [29], [32]. Here, we describe the development of a bi-sex RIDL system in pink bollworm using *piggyBac*-mediated germ-line transformation.

## Results

**Figure 1:**
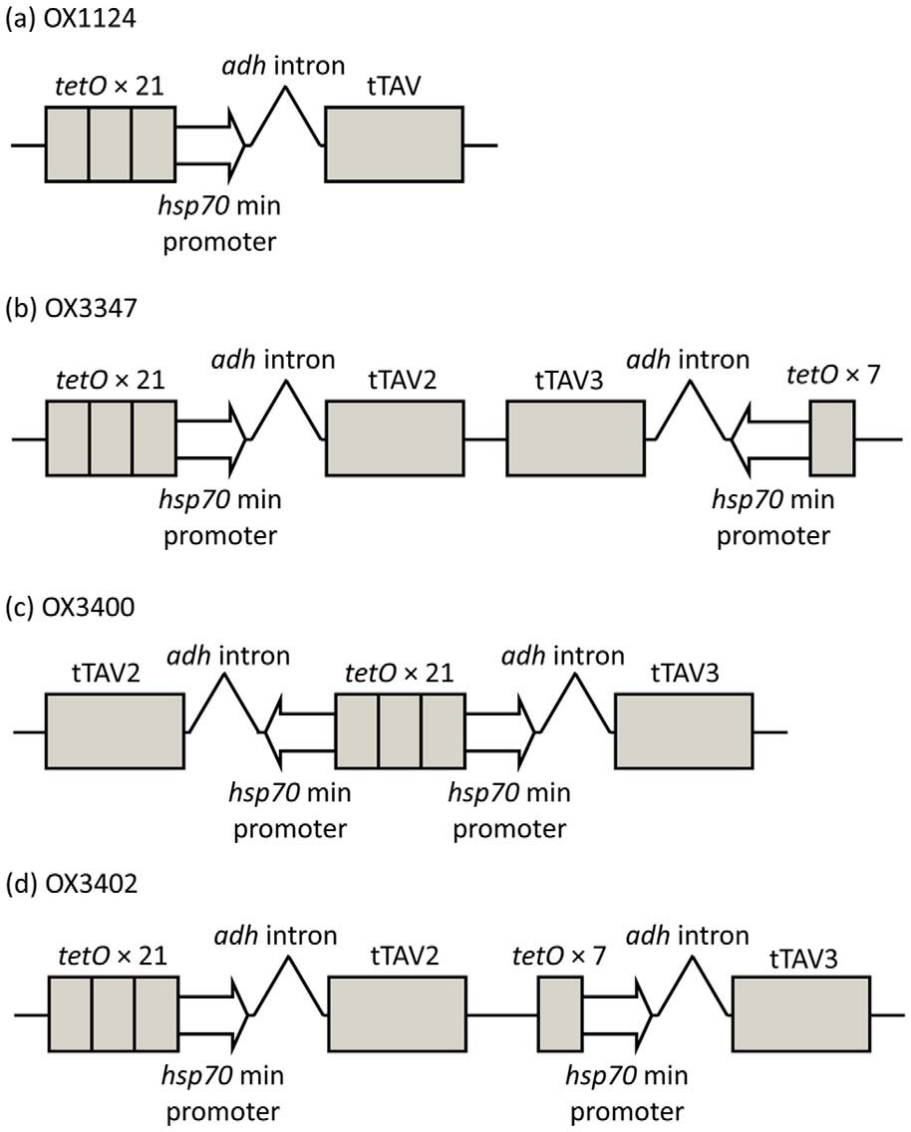
Schematic representation of the RIDL cassette (markers and *piggyBac* sequences omitted) of constructs used for transformation of pink bollworm: (a) OX1124; (b) OX3347; (c) OX3400; and (d) OX3402.

Following a similar design to the construct used to generate RIDL strains of *Ae. aegypti* [19] and medfly [18], we generated the construct OX1124, which carries a fluorescent protein marker and a cassette designed to induce repressible lethality. The latter comprises 21 repeats of the tetracycline operator element (*tet*O) linked to a minimal promoter, to drive expression of tTAV (Figure 1a), a variant of the tTA transactivator [33] codon-optimised for expression in *Drosophila melanogaster*. tTAV acts as a transactivator for *tet*O. This cassette is intended to form a positive feedback system that, in the absence of tetracycline, results in high and lethal levels of tTAV expression [18].

**Figure 2:**
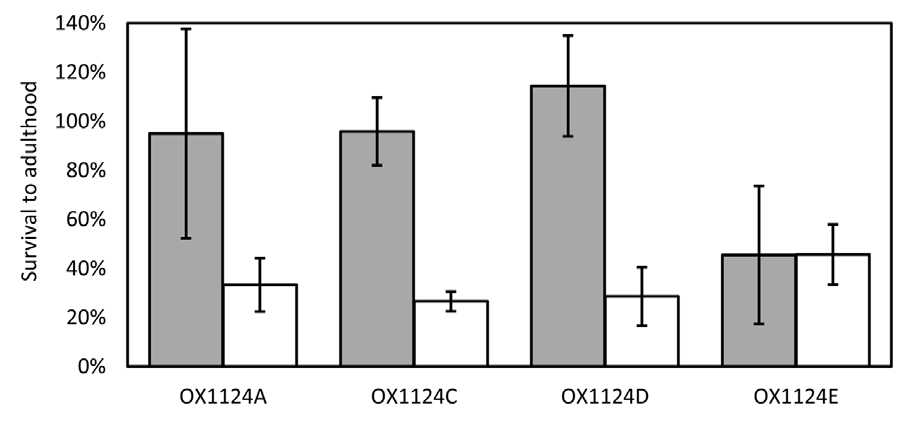
Survival to adulthood of transgenic pink bollworm, heterozygous for an OX1124 insertion (line A, C, D or E), reared on larval diet with chlortetracycline (grey bars) or without chlortetracycline (white bars). Survival is expressed relative to that of each group’s wild-type siblings. Error bars indicate 95% confidence intervals.

Four transgenic strains of pink bollworm carrying independent insertions of OX1124– named OX1124A, OX1124C, OX1124D and OX1124E – were generated by microinjection. Each line showed strong expression of the DsRed2 fluorescent marker throughout the bodies of larvae, pupae and adults. Each exhibited Mendelian inheritance of the transgene consistent with a single-copy autosomal insertion. To test for tetracycline-repressible lethality, heterozygous males were crossed to wild-type females. Their progeny were reared either on larval diet containing 100 µg/ml chlortetracycline (CTC) or on equivalent diet with no CTC. On CTC diet, three of the strains (OX1124A, C and D) showed good survival (87–96%) to adulthood (Figure 2), whereas OX1124E showed poor survival (45%). In the absence of CTC, the survival rate was much lower for OX1124A, C and D (27–33%). Survival of OX1124E insects was similar to that on CTC (46%). The moderate levels of CTC-repressible lethality seen in OX1124A, C and D demonstrated the function of the RIDL system in pink bollworm, but we judged that survival was too high off CTC for the strains to be further assessed for field use. The low survival of OX1124E insects on or off CTC indicated a dominant lethal effect induced by the construct insertion rather than by expression of transgenes on the construct.

**Figure 3:**
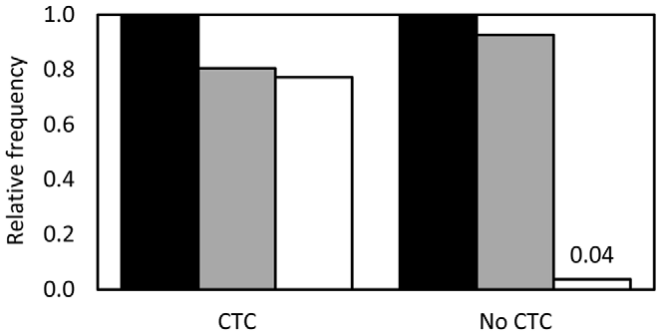
Relative rates of survival to adulthood of transgenic pink bollworm – heterozygous for OX1124A-only (filled bars), for OX1124D-only (grey bars), or for both insertions (white bars) – on diet containing chlortetracycline (CTC) or on diet without chlortetracycline (no CTC). Survival is expressed relative to that of OX1124A-only siblings.

We hypothesised that increasing the copy number of the *tet*O/tTAV cassette in the insect would amplify lethality off CTC. To test this, we crossed males heterozygous for both the OX1124A and OX1124D insertions, scored survival to adulthood of the transgenic progeny on and off CTC, and genotyped these adults by PCR to identify which OX1124 insertion(s) they carried. Survival of progeny on CTC was not significantly different between the three genotypes (p = 0.91): OX1124A-only, OX1124D-only and OX1124AD (Figure 3). Off-CTC survival was significantly different between the three groups (p = 0.01), with progeny carrying both insertions forming only 1.9% of the transgenic pupae. These results indicate that increasing the copy number of the RIDL cassette can result in a marked increase in induced lethality.

We therefore designed two constructs, OX3347 and OX3400 (Figure 1b–c), that carry two copies of tTAV-like genes – tTAV2 and tTAV3– with nucleotide sequences modified to reduce the risk of recombination when both are present, and TetR regions independently codon-optimised for insects. In OX3347, tTAV2 expression is regulated by 21 *tet*O repeats, as in OX1124; and tTAV3 is regulated by seven *tet*O repeats. In OX3400, tTAV2 and tTAV3 are in inverse orientation, sharing the same 21 *tet*O repeat sequences, which have bi-directional activity.

**Figure 4:**
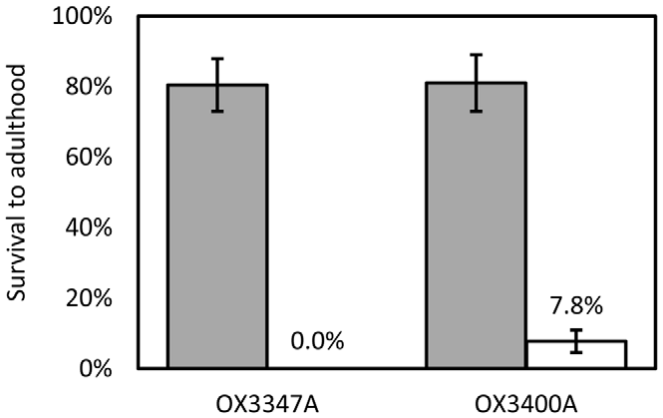
Survival to adulthood of transgenic pink bollworm, heterozygous for the OX3347A or OX3400A transgene insertions reared on larval diet with (grey bars) or without chlortetracycline (white bars). Survival is expressed relative to that of each group’s wild-type siblings. Error bars indicate 95% confidence intervals.

Microinjection of these constructs yielded one transformed line for each, named OX3347A and OX3400A. These were subjected to survival tests on and off CTC (Figure 4), as for the OX1124 lines. Both lines showed good survival to adulthood on CTC diet (80–81%). In the absence of CTC, no OX3347A progeny survived to adulthood, whereas 8% of OX3400A progeny survived to adulthood. With respect to potential field use, these data indicated highly promising phenotypes, particularly that of OX3347A. However, this insertion proved to be recessive lethal and homozygous lines could not be generated.

**Figure 5:**
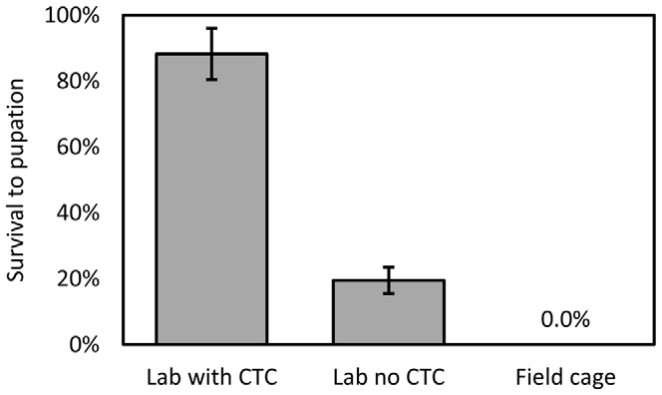
Survival to pupation of OX3400A pink bollworm reared in the laboratory on larval diet with or without chlortetracycline, and on cotton bolls in field cages. Survival is expressed relative to that of each group’s wild-type siblings. Error bars indicate 95% confidence intervals.

OX3400A was subsequently subjected to further test crosses in the laboratory as before, but a proportion of the eggs collected were also placed on host plant material - cotton bolls - in a field cage. In the laboratory, the resulting survival to pupation was similar to that seen in the survival-to-adulthood experiments. With no OX3400A survivors in the field cages (Figure 5), however, survival was significantly different to that of the off-CTC laboratory tests (p<0.01). These results indicate that, in pink bollworm, field conditions amplify the deleterious effects of RIDL without CTC. Those OX3400A larvae that might otherwise survive in the laboratory presumably succumb to the cumulative effects of high-level tTAV2/tTAV3 expression and environmental stress.

**Figure 6:**
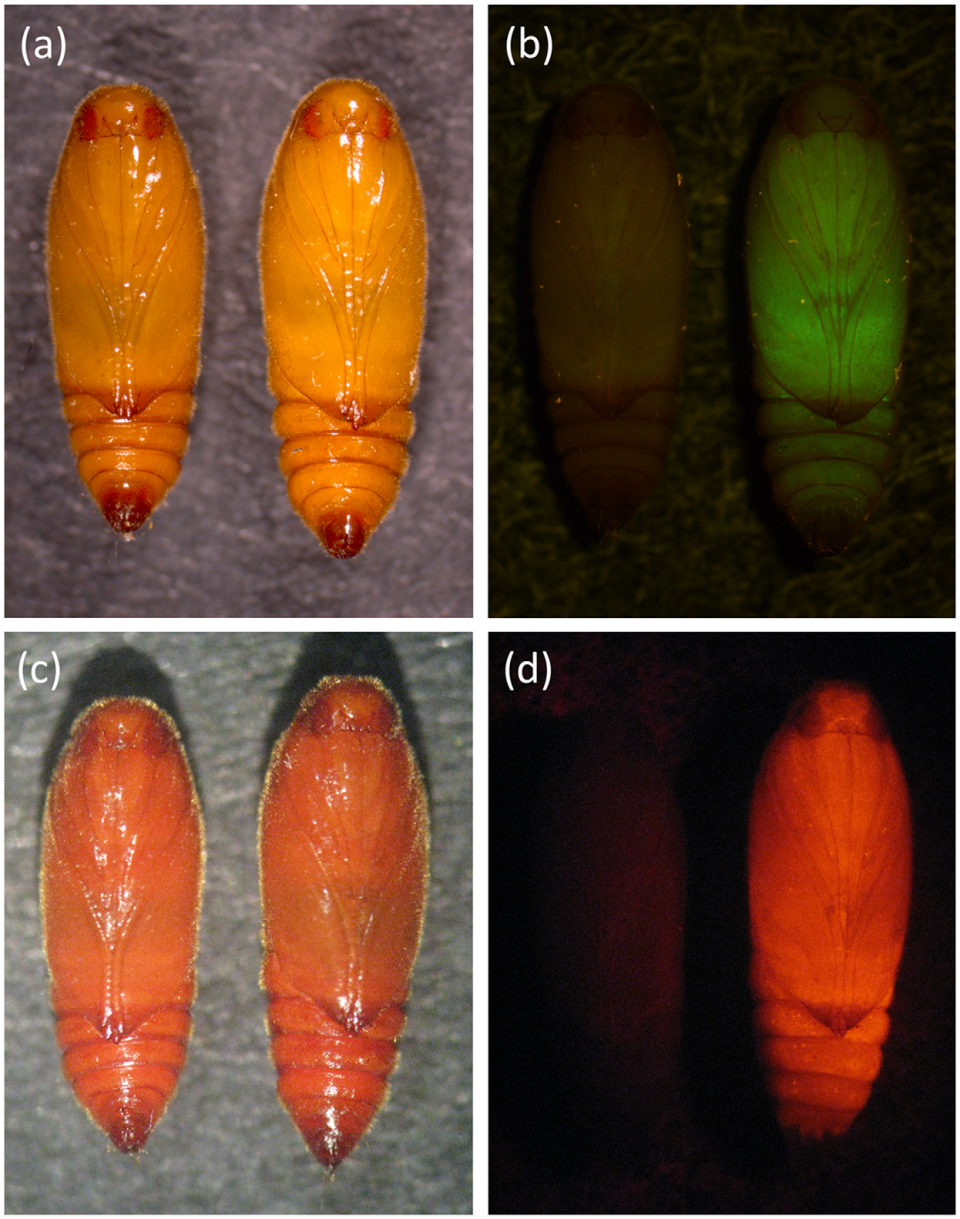
Fluorescent phenotypes of OX3347A and OX3402C. Photographs showing a wild-type pupa (left) and an OX3347A pupa (right) under (a) white light and (b) green fluorescent protein (ZsGreen) excitation wavelength light; and a wild-type pupa (left) and an OX3402C pupa (right) under (c) white light and (d) red fluorescent protein (DsRed2) excitation wavelength light.

**Figure 7:**
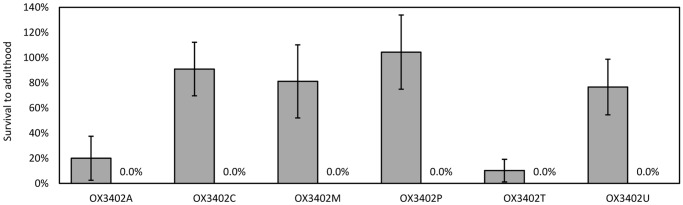
Survival to adulthood of transgenic pink bollworm, heterozygous for an OX3402 insertion (line A, C, M, P, T or U), reared on larval diet with chlortetracycline (grey bars) or without chlortetracycline (not visible as all data points are zero). Survival is expressed relative to that of each group’s wild-type siblings. Error bars indicate 95% confidence intervals.

**Figure 8:**
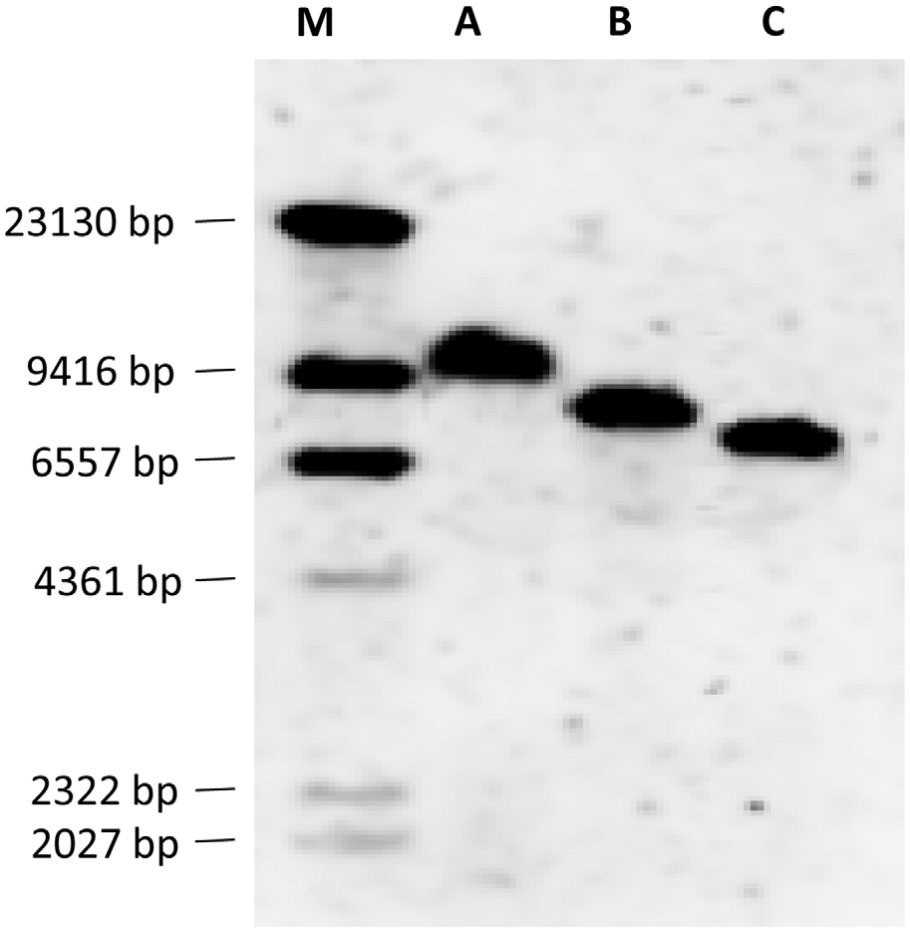
Southern blot analysis of OX3402C. From left to right, lanes are: Dig-labelled DNA molecular weight marker II (M), OX3402C genomic DNA digested with *Bgl*II (A), *Nhe*I (B) and *Xho*I (C).

The construct OX3402 is similar to OX3347 (Figure 1d) but carrying tTAV2 and tTAV3 in tandem orientation and a different fluorescent marker (DsRed2 instead of ZsGreen). OX3402 was microinjected into pink bollworm embryos with the aim of generating transformed lines showing repressible lethal phenotypes comparable to that of OX3347A. Six stable transformant lines were recovered: OX3402A, C, M, P, T and U. Their transformation marker showed red fluorescence that was more easily screenable than that of OX3347A (Figure 6). In test cross progeny (Figure 7), on-CTC survival rates were variable, with only OX3402C, OX3402M and OX3402P showing survival rates higher than 80%. All OX3402 strains, however, produced no transgenic adult survivors off CTC. In other words the dominant lethal phenotype in these strains is fully penetrant in the absence of the CTC repressor, even under laboratory conditions previously shown to be significantly more permissive for survival than field conditions. The two strains that showed the highest survival on CTC, OX3402C and OX3402P, were therefore selected as candidates for further development towards eventual field use. As with OX3347A, the OX3402P insertion appeared to be associated with a recessive lethal mutation. We were therefore only able to generate a transgene-homozygous colony of OX3402C. Subsequent Southern blot analysis of OX3402C indicated the presence of a single transgene insertion in this line (Figure 8).

**Figure 9:**
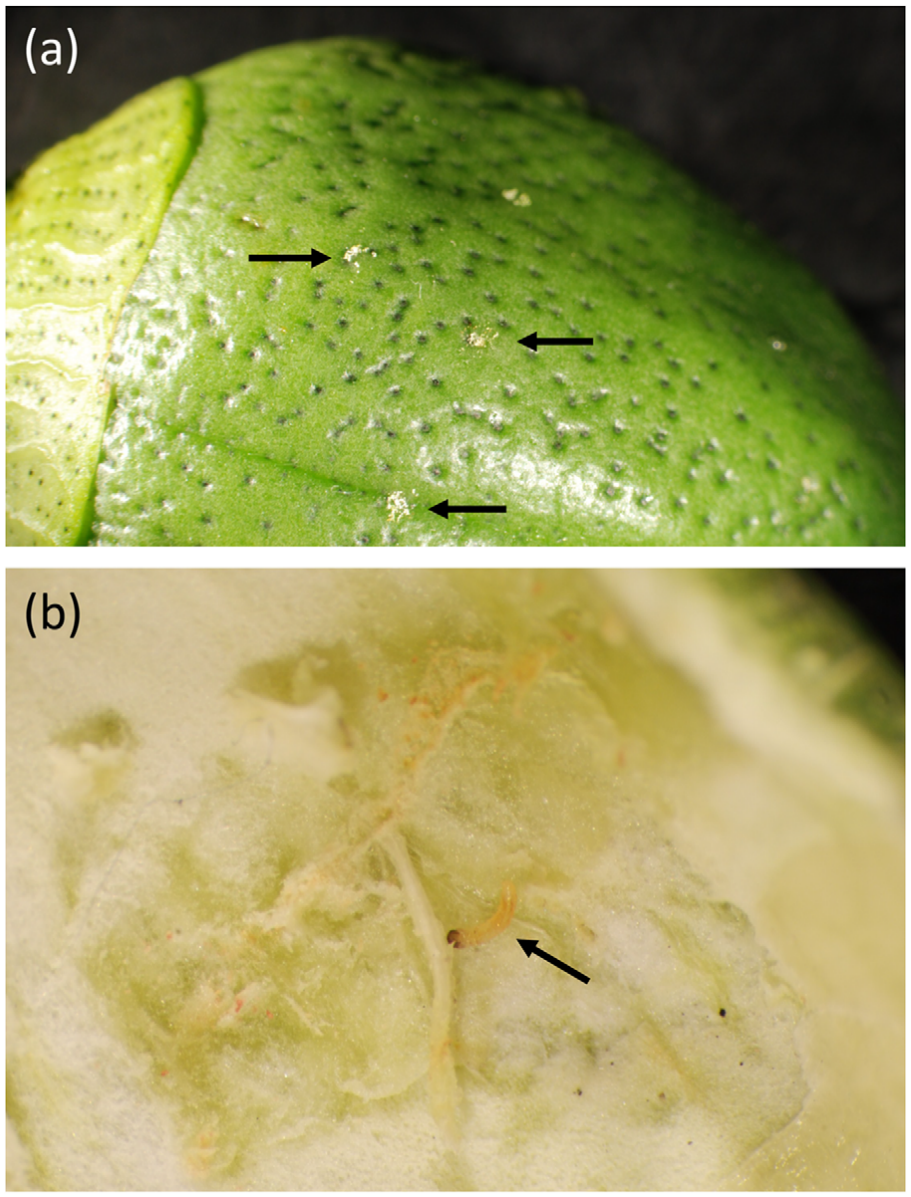
Impact of OX3402C larvae on cotton bolls in the laboratory. (a) typical marks on exterior of cotton bolls (indicated by arrows) caused by OX3402C larvae, and (b) minor damage to lint caused by a OX3402C larva that had penetrated the boll (indicated by arrow) but remained stunted at approximately 1 mm in length. Equivalent wild-type larvae had pupated at this time, after reaching approximately 10 mm in length.

In light of these test results, we concluded that if OX3402C were to be released over cotton fields their progeny would die in the absence of tetracycline or similar analogues. If this mortality were to occur late in development, for example in the final instar, the larvae may cause significant damage to crops before they die. On artificial diet the larvae tend to burrow as they feed, so time of death off CTC was difficult to assess in laboratory tests. We therefore set out to assess directly what damage OX3402C larvae, both homozygous and heterozygous for the transgene, cause to cotton bolls on plants. Cotton plants were infested with eggs – either wild-type, OX3402C-heterozygous and OX3402C-homozygous – and the bolls were assessed later for damage from larvae feeding and emerging to pupae. Wild-type larvae caused feeding damage to boll lint and seed, and pupae emerged. With both heterozygous and homozygous OX3402C larvae, small entry holes were evident, but no pupae emerged. Dissection of the bolls showed some minor damage to lint, and a small number of stunted larvae, some dead and some alive but inactive (healthy pink bollworm larvae tend to feed constantly and behave defensively when exposed) (Figure 9).

**Figure 10:**
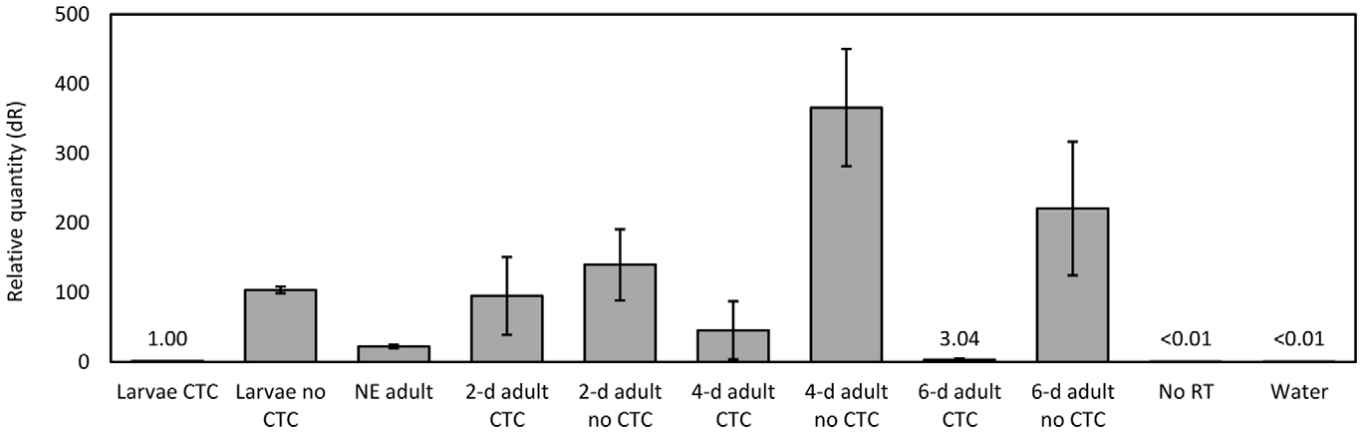
tTAV2 expression in homozygous OX3402C pink bollworm. Amount of tTAV2 mRNA in extracts from larvae and adults, determined by real-time PCR. Data are normalised to the level of tTAV2 RNA in OX3402C-homozygous larvae reared on CTC. Analysed samples were second-instar larvae fed CTC or non-CTC diet, and the following homozygous OX3402C adults (reared as larvae on diet with CTC): newly eclosed adults (<12 h after eclosion; ‘NE adult’); 2-day-old adults with CTC or non-CTC sugar water; 4-day-old adults with CTC or non-CTC sugar water; and 6-day-old adults with CTC or non-CTC sugar water. All samples underwent three technical replicates. For adults, biological replicates consisted of three single moths per sample category. For larvae on and off CTC, which each comprised one pooled sample of >10 larvae, technical replicates were used to calculate mean relative quantity. In adult samples, mean relative quantity between technical replicates was used to calculate mean relative quantity between biological replicates. No RT, no reverse transcriptase control reaction; dH_2_O, water control. Error bars represent standard error of the mean (between technical replicates for larvae on CTC; between biological replicates for adult samples).

On artificial diet, OX3402C-homozygous larvae also show stunted growth in the absence of CTC, prior to premature death. Quantitative real-time PCR indicates that tTAV2 expression is around 100-fold higher in second-instar OX3402C-homozygous larvae reared on non-CTC artificial diet than in those reared on CTC (Figure 10). This tightly regulated expression is similar to that seen in a similar RIDL strain of medfly [18], which also showed higher levels of expression from two copies of the transgene than from one, as expected. tTAV2 expression in OX3402C adults fed non-CTC sugar water can be >3.5-fold that of larvae fed non-CTC diet, with no obvious sign of ill health in the moths. Clearly, the effect of high tTAV2 expression levels (and presumably those of TAV3) on insect mortality varies according to life stage, with larvae much more susceptible, as also seen in medfly and mosquitoes [18], [19].

## Discussion

The transformed strains described here demonstrate the function of a tetracycline-repressible lethal system in pink bollworm, the first non-Dipteran insect in which such a system has been constructed. Furthermore, the engineered phenotype described in OX3402C indicates a strain with potential for control of wild populations of pink bollworm. Previous assessments have shown that transgenic pink bollworm, irradiated, can perform well in the field compared to a wild-type counterpart [29]. However, the use of an un-irradiated RIDL strain, such as OX3402C, could provide several further benefits. Field trials may seek to determine whether any performance costs induced by the transgene insertion, as possibly indicated by a survival-to-adulthood deficit described here, are greater than that induced by irradiation and associated handling in conventional SIT. The fluorescent, heritable marker would provide certainty for SIT programme monitoring. The 100% mortality of OX3402C in the absence of CTC indicates that it could not reproduce or establish in the wild. These traits combine to provide verifiable bio-containment. With a RIDL strain such as OX3402C, accidental releases of fertile moths - which have been reported in pink bollworm SIT [34] - would be self-limiting and without serious pest consequences for growers.

OX3402C larvae caused little damage to cotton bolls in the laboratory, and we would expect this impact to be even lower in the field. As indicated by the lower survival of OX3400A in the field cage compared with the laboratory, the greater variability of field conditions might be expected to reduce the time-to-death of OX3402C larvae, with consequent negligible crop damage. Further confirmation of this effect will be required in future field experiments with OX3402C and similar lines. In addition to field performance, assessment of a strain’s life history parameters is needed, to determine how efficiently male moths can be produced in large numbers under mass-rearing conditions. The high levels of tTAV2 expression in OX3402C adults supplied with non-CTC sugar water – higher than that seen in sick larvae on non-CTC diet – may have a negative impact on adult performance. However, high-level expression of tTAV seems relatively innocuous to adult medfly and *Ae. aegypti* mosquitoes [18], [19], [35]–[38]. Testing OX3402C longevity will help to identify whether this may reduce their effectiveness in the field.

Cotton has been at the forefront of plant biotechnology, with transgenic cotton now comprising 60% of global cotton production [39]. The majority of these strains express *Bacillus thuringiensis* toxins (*Bt* cotton) to confer resistance to lepidopteran pests. With resistance to *Bt* cotton developing with pink bollworm field populations in India [40], resistance management is a growing priority for growers. Resistance to SIT also a theoretical possibility, but has almost never been seen in more than 50 years of practice. One instance of behavioural resistance was seen during the successful elimination of melon fly in Okinawa [41]. The possibility of resistance to RIDL has been explored *via* modelling [42], but no actual resistance has been observed. Indeed, conventional SIT has provided resistance management for *Bt* cotton in south-western USA [43], in place of the standard practice of maintaining non-transgenic cotton refuges. OX3402C or similar strains could fulfil this role where radiation-based SIT against pink bollworm is not feasible, for example where the costs and security issues of establishing irradiation facilities are prohibitive.

The engineered pink bollworm described here may permit extension of SIT-type control to cotton-growing regions where SIT is not available. Field demonstration of the efficacy of OX3402C in suppressing a wild population might represent the next step of development of this technology in pink bollworm. Furthermore, development of this technology in pink bollworm paves the way for similar work in other major agricultural pest Lepidoptera. To control them, growers are still highly reliant on insecticides where transgenic crops are not available. Alternative tools, such as RIDL, are becoming increasingly valuable in the face of declining consumer acceptance, development of pest resistance and other difficulties associated with chemical control.

## Materials and Methods

### Plasmids

All constructs described here are non-autonomous *piggyBac* elements, i.e. incorporate terminal sequences of the *piggyBac* transposable element. In canonical transposition events, only the DNA segment flanked by these sequences inserts in the insect genome and only in the presence of exogenous *piggyBac* transposase (‘helper’) provided separately [32], [44]. OX1124, OX3347, OX3400 and OX3402 all carry a fragment of the *immediate-early-1* (*ie1*) gene from the AcMNPV baculovirus together with the hr5 enhancer from the same baculovirus. In OX3347, this sequence drives expression of the green fluorescent marker protein, ZsGreen, and in the other constructs drives expression of the DsRed2 fluorescent marker protein (Clontech Laboratories Inc, Mountain View, CA, USA) [45], [46]. Aside from marker sequences, the constructs differ in the number and arrangement of components of the tetracycline-repressible system (Figure 1). Annotated plasmid sequences of OX1124, OX3347, OX3400 and OX3402 are archived online (http://www.ncbi.nlm.nih.gov; Genbank accession numbers JX242582, JX242583, JX242584 and JX242585, respectively [47]).

### Pink Bollworm Rearing, Transformation and Analysis

Pink bollworm were reared on artificial diet supplied from the mass-rearing facility in Arizona, USA, using methods modified and scaled-down from standard pink bollworm rearing protocols [48]. The wild-type strain, known as ‘APHIS’, currently used in the US sterile insect technique programme was used for transformation and as the wild-type comparator strain in these experiments. Larvae were fed with the mass-rearing diet, which contains 100 µg/ml CTC, an analogue of tetracycline. CTC was withheld from the larval diet for off-tetracycline tests, in which constructs were designed to confer de-repressed lethality in transformed insects. Transformation was performed by microinjection, following the method of Peloquin *et al.* [32]. Each insertion was derived from independent groups of G_0_ parents. Where possible, the flanking sequence of each independent transgene insertion was determined following the method of Martins *et al.* [49] (Table S1).

For test crosses, eggs were collected from parental cages, divided and the fragments placed on CTC or non-CTC diet. Pupae were collected, scored for the fluorescent marker and counted. The pupae were held in Petri dishes where they were allowed to eclose. The numbers of resulting adults from each genotype were also recorded (for raw data, Table S2).

Similar experiments were conducted to compare survival of OX3400A larvae on laboratory diet and on host plants in a field cage (USDA permit number 05-118-01r). Laboratory tests were conducted as above, but a fraction of the eggs were also placed under the calyx of cotton bolls (29 bolls in total, short staple variety AG 3601) enclosed in an organdy sleeve cage and grown in a screened quarantine cage (3 m×3 m×2.5 m) located in Phoenix, Arizona, USA. The bolls were brought into the lab after 1–2 weeks in the field cage and placed in separate containers to catch larvae exiting the bolls. These pupae, and those from the artificial diet, were screened for the presence or absence of the fluorescent DsRed2 marker and counted (for raw data, Table S3).

In the laboratory assessment of larval damage on host plants, mature cotton plants (Pima variety, *Gossypium barbadense* L.) carrying bolls were moved from a greenhouse into the quarantine laboratory under grow lights. Eggs were collected on paper from three cage types: one containing OX3402C-homozygous male and female moths; one with OX3402C-homozygous males and wild-type (APHIS) females, and the other with wild-type (APHIS) males and females. These cages produced OX3402C-homozygous, OX3402C-heterozygous and wild-type eggs, respectively. Pieces of paper carrying 10 eggs of one of these genotypic classes were attached to a boll with tape. Plants were maintained at 30°C, 50% relative humidity, and constant light. Plants infested with each egg type were held in separate incubators.

### PCR Genotyping

The genotypic class of different moths was determined by PCR [2 min at 94°C, 3×(10 s at 95°C, 1 min at 58°C, 2 min at 72°C), 32×(10 s at 95°C, 30 s at 57°C, 55 s at 72°C), and 5 min at 72°C]. To detect a specific transgene insertion, two primers were used: one specific for the flanking sequence of the insertion; and one specific for the construct sequence (Table S4). To determine copy number of a given insertion, primers specific for genomic sequence from each side of the transgene insertion were also used: in the absence of the insertion a specific fragment was amplified; in the presence of the insertion the distance was too great for amplification under normal PCR conditions, and no such fragment was amplified. In insects heterozygous for a given transgene insertion, both the former and latter fragment types would be amplified. In transgene insertion-homozygous insects, only the former fragment type would be amplified.

In order to establish a transgene-homozygous colony of a given line, these PCR analyses were conducted using genomic DNA extracted from single hind legs removed from an anaesthetised moth [50]. The OX3402C-homozygous line was derived from 62 homozygous founder moths.

### Southern Blot

Genomic DNA was extracted from pink bollworm pupae from the OX3402C line using proteinase K and phenol, as described by Sambrook and Russell [51]. Samples of 5 µg genomic DNA were digested with three different restriction endonucleases that each have one restriction site within the OX3402 construct (*Bgl*II, *Nhe*I and *Xho*I, Thermo Scientific). Digested genomic DNA and a digoxigenin-labelled molecular weight marker (DNA molecular weight marker II, Roche) were separated by electrophoresis through 0.7% agarose gel and then blotted to positively charged nylon (Roche) membrane by capillary-transferral [51]. A DNA probe was synthesised by PCR from DsRed2 plasmid template with DsRed2-specific primers using PCR DIG Probe Synthesis Kit (Roche). Detection of hybridised DNA-fragment was performed using DIG High Prime DNA Labelling and Detection Starter Kit II (Roche) and UVP ChemiDoc-It 500 System.

### Quantifying tTAV2 Expression

Total RNA was extracted using TRIzol (Life Technologies) according to the manufacturer’s instructions. RNA was treated with DNase (Qiagen) to eliminate genomic DNA. 0.5 µg of RNA was converted to cDNA using RevertAid First Strand cDNA Synthesis Kit (Fermentas), with oligo dT as the primer, according to the manufacturer’s instructions, 1 µl cDNA was used in subsequent real-time PCR.

Quantitative real-time PCR was carried out on Mx3005P (Stratagene) and analysed with MxPro software (Stratagene). Assays were carried out in 25 µl total volume with 1×TaqMan Master Mix (Applied Biosystems), 0.4 µM forward and reverse primer, and 0.2 µM probe. tTAV levels were determined using primers tTAVrealF and tTAVrealR, and probe tTAVFAMP (Table S5). Expression levels were normalised to tropomyosin, which was amplified and detected using primers PBWTroF and PBWTroR, and probe PBWTroP (Table S5). Real-time conditions consisted of an initial denaturation of 95°C 10 min, and 45 cycles of; 95°C 3 s, 60°C 1 min, and 72°C 20 s. Fluorescence was collected at 60°C.

### Statistical Analysis

One-way ANOVA was used to assess the impact of OX1124 construct copy number on survival to pupation. Comparison of OX3400A mortality in the laboratory and in the field cage was conducted using a two-proportion z-test (Microsoft Excel 2010). Calculation of 95% confidence intervals shown in figures for mortality ratios (standardised ratio, SR) was conducted using the following formula: CI = SR ± (1.96×SE); where SE = (SR/square root of number of observed events) [52].

## Supporting Information

Table S1 **Nucleotide sequences of the genomic regions flanking each transgene insertion.**
(DOCX)

Table S2 **Survival to adulthood of transgenic and wild-type progeny of transgene-heterozygous males crossed with wild-type females.** Progeny were reared on diet with or without chlortetracycline (CTC and non-CTC, respectively).
(DOCX)

Table S3 **Survival to pupation of transgenic and wild-type progeny of OX3400A-heterozygous males crossed with wild-type females.** Progeny were reared on diet with or without chlortetracycline (CTC and non-CTC, respectively), or on host plant material in a field cage.
(DOCX)

Table S4 **Sequences of primers used for PCR genotyping of strains OX1124A, OX1124D, OX3347A and OX3402A (sequences shown 5′ to 3′).** The primers specific for peripheral sequences in the construct were used for all strains.
(DOCX)

Table S5 **Sequences of primers and probes used for real-time PCR of OX3402C samples to quantify tTAV2 expression (sequences shown 5′ to 3′).**
(DOCX)
