# Supplementary material for: Engineered Repressible Lethality for Controlling the Pink Bollworm, a Lepidopteran Pest of Cotton
Source: PLoS One. 2012 Dec 4;7(12):e50922. doi: 10.1371/journal.pone.0050922 (PMC3514271; doi:10.1371/journal.pone.0050922)
Supplement: Table S1 — Nucleotide sequences of the genomic regions flanking each transgene insertion. (DOCX) [file pone.0050922.s001.docx]

| **Strain** | **Nucleotide sequences (5ʹ to 3ʹ; bold text indicates construct sequence)**  **TTAA target site duplication is underlined** |
| --- | --- |
| OX1124A | **5’ end**  ACAAAACCTTTTTCCCCATGGTTTAAAAAAGGTTGGAAAATCACGTTAGACTCCGACTATGTTAACTGCTTCGAGAGATGTAGAACTCTTCTCACTAATGACCCCATACTTCAGTGGCCCTGNCTTCATTAA  **3’ end**  TTAATAAGGATATGTCCTGAAAACATTGAAAATCCAACATACATCACCGAGACTATTGGTGCTATGATTTTCATCGTTTTTTGTCACTTCACTGCCACAAGACATTATGTTAACAAAGTGTCCATTGGGAACTTTCATTTTATTCACGTCACTTTACACTTAAACTAATACAAAACCCGTTAAAATGTAATAATCGACCGATCGCAGAAGTATAAACGACAACGACAAGTCAACATCGATCGACTGACATTAAAAAAACCTTGCCACAATTTTCTCAGAAATCTACCAAAATTAATTGTTTGTTTCCTGAAAAGTGGCAACAACAACCAAACTAAATCTGTTTTGAATTTTCCCGCTTATTTAATACTTTATTCTGATTGGTTGTTGTGGTGATAGACAAATTTATTCTCATTTCCTATTGGTTTCTTATTAAATTCTCAATGGCAACGGGAACAAGATTTTTTTCTGTGTCAAACATTCATTCATGGCGTGAGAACGAAGTGAAACGTGTTTTCGTTTGTTCGAATTATTTGTTTTTGCTGGAAACCACGTGTGAAATCTATCAGAAAAATATTCTAAATTAGAGCTTCTAGGTTTTATAACATTCCTATTGTAGAATAAACTGACCGTTTTTGTGTGAAACCTTGTGAAAATGGTGGTAGTATCGTC |
| OX1124C | **5’ end**  TCGGTGGCCTGGCTCTATACTTTTCTTCATTCTCGCAATGAACCCGGTATGACGCGATACGACTGCGAAATGCGCTGGTTATCTTTTCTATACCTTCACATTCTATAATAGAGCTGTTATTTTTATGCAAAATACTTCGCAAATGAGCGGAAAAAGCTTTACTTTTTATAGATAAATCGCAAACTGAACAATAAACGGACCTGGTAACTTTAGACATCTTGAGGACAATAAGGAGCTATTACAATATAATTTAAAAGTTAAAACTAAACCAAAGAGATGTAACTAAAGAGATGTTTCTCCTTTAAAGTTCAAGATATTTGCTTGTCGAATATTCGAACGGCAGAGTCGCAGCTCAGTTAGTTTGTTAGATAGTTATGTAAATATTAGAAGAAGAAAATATAGTGAGTGTGTATTGATATATTTTCCGGCGAGGGATATCTAACTTTTATTAA  **3’ end**  TTAATAAATTATAATGCGCTAATAGAATTCAAAACTATTTATATTATATTTCATAGAACCCTAAAACACATATCTAGCATTAAGTCACTAAAGCAAATTAAGACAATTTTAGAAAAATAAAACATGAAAGTTTAAAATTAAAGTAGTAAATAAGTAAGATGGCATTTAAATGTACGGGTACGTGAATAACCGTGAGAAAGCGAATTTCATTTCTTATTTGCGAAAAAAACACCTTCTTCTGTAAATTGCTTCTTACAGCATCGGACAAATTCTCGTAACGGCACCAGCTCCGGCGCGTCGTTATCTGCTTGATACTTTAAGCTTAAAGTCGCTTTCTCCCACCGGTCCTTCGGCTGTACCTTACGTATGTCTTTGATATTGTAGAGCCTTAGCTCTAC |
| OX1124D | **5’ end**  GACGACAGAAAGGGCGTGGTGCGGAGGGCGGTGCGAAAAAGTATATAAGAATTTTGGTGTAAGTAGAGAGCATTATAAAGTCATATAAACATCACCAACTTGTACCAGCTGATATCCCTCATAACCTACCTAGTAAATATGCGATGTTGACTTCAATAAAAGTTTTATAGATTTCAATATTTACATTTGATTAGATTAGGTATTAGGTAATCTTGTACGCGCTCAGTTTTTANCCTGCGAATTCTACAAAAGAACAAATTGTTGGGTTCTGTTTCAGTGATTTTTATTAAAATAAAATGAAATTTTATTAA  **3’ end**  TTAATAAGGATATGTCCTGAAAACATTGAAAATCCAACATACATCACCGAGACTATTGGTGCTATGATTTTCATCGTTTTTTGTCACTTCACTGCCACAAGACATTATGTTAACAAAGTGTCCATTGGGAACTTTCATTTTATTCACGTCACTTTACACTTAAACTAATACAAAACCCGTAAAATGAATAAT |
| OX1124E | **5’ end**  GATGTCCTAAATGCACGTGTAGCGTGAAGACGACAGAAAGGGCGTGGTGCGGAGGGCGGTGCGNAATTTGAAGTTACAAAAATACATTGCGATTTCGAACTGGCTACAATTAATGCCATCAATAATGTGTTTCCCAATGCGAAAATTATAGGTTGTTATTACCATTGGTGTAGAACTGTATGGCGTAAAGCAAAACGTTTAGGACACACGAAAAGTAAGGCAGAGAGGAGAATTGTAGCTCTAACTGCGGCGCTTTCACTTATCCCAGAAGAAAATTTTCGCGAGGGCTGGTCTTATATTAAAGCAGAGTGTGGAGATGAAATTCAAATGCCAAAATTTATTAACTACATACAGCGGTTTTGGATAAACAAGTCGGTATTCGAACAACATTCCGCATCCAAAACGACGATGTTATCACACGTGTACAAATGCAATTAGTTAA  **3’ end**  Not determined |
| OX3347A | **5’ end**  TGACGTCAGAAAGGGCGTGGTGCGGAGGGCGGTGCGATATCTCGTCAGAATAGTTCCAGTTGTGCCGAAACAACTTCAACCGCAAAATGAATGTTCAAGGTTGTTACATTATTTAATTTTACTGTTTTTCATAGGTGTCAGTTTGTGAGAAGAGTATGATATTGATGCCGAAACCTTTACTGAACAATGGATGGCGTTCAGTTTGACACATCTGAATGGTGCTGCACCGACAGTTGAAAACTTGGATTTGCTTGCGAGAAAGGAATTTTCCAAGCGGGCGGCGAGTCGCCTCAACGCGCCCGCCAAAGATGCGGCTAGATCAAGTACCGGCTCAAGTTTGAAGGTTTATGGCGCGCCTGTTTCTGCTCAGTATCCTTTTATTACGGTTCTTCCACGTGTTACATTGCTTGAAATGTATCATTTTATACTTATAAACTAGTAAACAAGAAACGTACTTACCTGGAAAGTACTAAAATGCTAGTGAGCGACATGATTACATGATATAGCCATTGTGTGTGTTTTCATGCAAATAGACTTTGTTCTACGCTTTTACAGGTTATGTTTTATTGTACATAATAAGCCTTAAAAATTAGATCAGATAATAATGTCCTGTCAAATTACATTGCTGCTCC*CAAGGTAAGTTGTCTGAAGTTGAGGT*TATATTATTTAGAAGATGAAGTTATGTGATTATAGTATGAAGAAATACTGAGGAGTACTAATCACTAACTACTGCTACCACTTCAGTTACTTGTCATGTGTCTTCTTATGAAATCTGGATGCATTTGAAGAGAAAGGACCTTTCTACCTGTGCTAAGTGAGTGTAAGTCTAGATGCTCAGCTCCCTACTGTGACTAGGTCTACACTCATTGAGTGCTTGTAGAATAGACATCTGAAGGCAGCAGCAAGAAGAAGATGAATAAATTGTATAGTTTTGATTAATAAAAGTGGTTTGTATAAATGTGATTTGACTTGATGATATTTGTGGGCGAGTTTGCTTCTGGTACATGTTCGCTATAAATCATGACTAGATCCATACTCGTCTGCAGCCATCAACTCAATCGCCAGTCATAATCAACTATGGTTATTTAA  **3’ end**  TTAAAAAAGTCTCACATGTTTTAATAATTTGTTGTGAGGGTAGGCGAGCCAAAAGTTACTAAGCATGATAGTATTGTTAAAAATAAGTCTATGCTTAGTAAATGTTACTTTAAATGCAGTGATATACAATTAATCAAAGTGTTATAAGTAAAAAGACAAAAAAAAAGCATAAAAAAATATACACCAATATATTATTATTACTCTTATTATTTTATAGATAAGGTATCTATGCCTACAGCTTACATAATTATAACACATTAAACTAGATATATGTAACTAAACTTTATAGAAGTACACATCATTTTCCTATATCTAATTTGAAAATGGTAATAATGATTTTTAATGTATTTTTTTTTTCACTAATCAATGGCTGTACATACATAACCTTTTCTAGATATACAAATTGTTGTTGTTTAGAACTTTATTGTTGTTGTTGTTTGGGACTTTCTAGGCTACATTCCCTAAAAAATAGATGGAGCGGTAAAAAACTCCTTCGTTTATTATTTCATGGATAACAAACATGCGTCTATTATCTTTGTATTTAGGTATAAATAACGACCCTTTTAATTACACCCAGGCACAATACATCTTTCACCCATTATTATTCTGATCCAACGCCCTCCGCACCACGCCCTTTCTGTCGTC |
| OX3400A | **5’ end**  GTGGCAAGGTCTACTGAGTACTACACTCTGTGTAAATACCATAGACTTAGAATAACTAAAAAAATTATAGACACAAACAAACTTCAAATAAAAACATGAATTTACTTAAGTATTTTCTTTAATTTTACATATTCGTGAAAATGTAC*GGTTCCAAAGTCAGGACTCATGATG*TTTTCCGGCCCTCGCCGTGCCAAGGAATACTAGTGGATATTTATTTGACAGCTTTCTTAAATTATGCGCGCGTTTGCGCCGCCTCTTACTTGAATAAAAACATCCCACTGTACCGACGGTACTTTATTTTGATTAAAATATATTTTCGTCGCGAGCAGGGCTAGA**TTAA**  **3’ end**  **TTAA**AAATTTAAAAATCGAAAGTTTTTGAAACTGGTTCTTGTATTTTGAAAACGTTTATGTTTTCATTTAGTTTTCATCTTTTATCATTGTTTAATAATTTCGAATTTGCGCGGGTAGGTACATGTATTTAAGTACATAATTTGTATAGCGGTATAAATTAGGTTATATAAATAGTTTAAGTCCGCGCCCTTAAATTAGGATTGTTAAAACAAAGAACAATATCGGTTGTACAATGTTGTCATTATCTAAGTATGTCACTGCGACTAAATTCGATCCACCGCCCTCCGCACCACGCCCTTTCTGTCGTC |
| OX3402A | **5’ end**  CTGCTGTCTTTCCCGCACCACGCCTCCCGCCACGCTGTTGTCCACCAACTAAAACTAGCGTTTACAGAAGTTGACGTTAGAGTTGTTAAAAATTTTGTTGAGAATTTATAAAGAACCGATGATGACATCCATATGCATGTAGATCCATACGTCCTATCGAAAGGGGAATGTACAAAGAACACTTAGGAGTTGTCGTTTTTCATTGACCGCAAAGAAGCCTAGTAAAATTATATCAATTATAATCCATGGCTTCACTTCACCTAAGTGTAAATTCAGCTCTGAATTGGGATCTTTCTATCAGACGCATTTTAA  **3’ end**  Not determined |
| OX3402C | **5’ end**  GCAAGATGACGACAGAAAGGGCGTGGTGCGGAGGGCGGTGCGGTATATAAACACTCTAGTATACAAAATGGGCAATATAACCTACCTACTCTTAATTAATAGGTAAAGGCCGTATGTAATAGTACAGAAGATACTACATAATTGTAATAATAATGTGGTTGTGATTCTACCTAGTAGTATATTACTTACCACACGATATTATCGAAAACCTAATTAACACAAGTCTTAATCAGGTTGGTAGTAATAGTAAGCTTAGTAATTATTTCTTATTATCTAAGCCCAACACAAATGCACACAGATTAAGTACAGCGTTCCGGCCGAATGCGGGATTTTGCTTATAACCACGGCGTATTTTTATGTGAACACGTTTTACAAAAAAATCCGAAACGATCCTAATACTATTTACCAATGTTTAGAACGAGCTATAAACATCTCATAGCATTTGATGCGATTGGCAGTTTGCATATAAATAACCCTTGCATAGGTAATTCTTGCAATTACTCCGCCCGTTATTTGAAATACCTACATACCTACGTACATTTTACTTCACAATAGCACACATCTGCATAGTATACGTATCACGTCTATAATCCCTAGGTACAGTAGGTACTAGGTACACCACACGTCAAGCAACAGCAACTGGCTTAA  **3’ end**  TTAACATAACAAATACTTTATTGCACAAACAGGAAAAAAACAAAATACAAAACAAAAGAGAAATAAAAAGTAGTATAAGTGGCTTGCTATTCTAAGAGCTAGCATTTTTATAATATGTAATAGTGCAAGAGTCAACGGTCGCCAGACAACGTAGGTCAGGCGACCGTTGACTCTTGCACTATTAGATCTTGTCT |
| OX3402M | **5’ end**  GACGACAGAAAGGGCGTGGTGCGGAGGGCGGTGCGAAATCTATGGACTCTAGTTAAAATAAAGCGCCCTCTATGAAAATTATAGAGAATCATATCAACCACGTCTCTGTTCTTACAGTACACTCATTTTCATTGTTTTAGAGAAAATGGTGCTACTCGCCGATAGATGGCGCTTTTTTAGTTTTTCTGCTCGCCGGTAGATGGCGAACAATGAGTTAATTTCCACCTTCCACATTACTTTTATTTAA  **3’ end**  TTAATATAACATTACAGAAACTACCTGATGTAAGTTACTTTGATATTATTTAATCAGAAAATAAGAACGTTATTACGGACTATTTTATAGAATCTATTTTGTATCATAATGATTCATCGTTATTTAGCTAAATGATGACTAGTTTTCCTAGAAAAATCTACACAAAGATATTCTTTTCTAACTCCCAATTATGGTCTACTAAACCGAATGCCAATACATTTCGTATCTTTACGTTATCAGTTAACGGCCATTGTTCCCAATCTAGAGAGAAACATAAATATGTACTCTCCGTCCACTTACGTACTCATTTATATGTTAATGTTCCAACATCCGATTCGTCGAGATTCATACACAGATATCTCTGGTTGATATCATTTTCACTGCCTTATTACTTTCCAATTTGTCTTGAGCATTGGTTATTGCCTCATTTATTGCGACCCCTACATCTAATAACTTCGCTTTGTGGACTTTTGTATATATCAAAAATATTCGTCGAACGCGAAAAATAATGGAGCAGCTTATATTTGTGATTCAGAATAAAAAGCTGGGTACTAATTATTAAAAGTGAAACCTTATGAAATAACTTAAGACGTTTAACAGTACCTTTTCGTCTTAAGTCGATGCCAGAGTCGAAAAATCTTGACTTACTCCTCGTAGACAGGTCCATTTTTTCAATAAATATCCACGATGAATTCACAAATGAGTAGATGAAAATAAAACTTAGTATTAGAAACTAAAACTAATGAAGTCTCTTTAACTTAAACTAAAACACTACACAAAATTAGAAGATTACAAAGTTCCAAGTCCGAGTAGACCACTGGGCACTGAAAGTCGAGCTCCTTGAAGTAATAAAGCATGAGATCCACCGCCCTCCGCACCACGCCCTTTCTGTCGTCATC |
| OX3402P | **5’ end**  GACGACAGAAAGGGCGTGGTGCGGAGGGCGGTGCGATATGCATTTTAA  **3’ end**  Not determined |
| OX3402T | **5’ end**  CCCTAATTTGGTTAGGATATTGCAAGCTCGGTGCTCGGAAAGCACGTTAAGCCATTGGTTCCGGTTGTATTTTTTTACTTATATAAGTACGTAGTCGATACTTGTCTGGGGCCTTTGGAAGCTCAGTATAGTAACCCTGACATAAAAATAGTTCAGTAGTTACTTATTCAGATATGTACACATGAATATTAGTAGAGCGTGCTTTTCGTGTACAAAAACGAGCCGTGCGTATTATCGCGCGTATTCGTAGCGATGTGTCTGCGCGTCCTTACTTTATAAAATTTAAAATAATGACGTTTCCGTGCTTACTAATATACCAGACTGCTCTTTATGCGTACAACAATAGAGAGTTGTACAGTTGCAGAGGTGACAGACGAGGACACGATGCTCGGCACGCGCATACTCGACGGGGTGCCTCATAGGCTCCGGAGGAGCGCGAAAACTGTGCATGTCATGGCCCCGGGAGTGTACAACAAACTGCCTGACCATATCATACAAGCAAAGAATTGTACTATATTTAAACTTAAACTTAAAAAATGGCTTATTGAGCAGGCATTCTATAGTTTTGACGACCTGTAATTGATAATATATTTTAATGTAATTGAATTCCGTAGTAATATTTAATTTCATTGAATTTTGTAATATCTGTGACGTGTAATTATTATTATCAATAAATGACTATGACTATGACTATTGTAACCCAGAATAGATTATTGACCCCCATAAATTACGTATTTAGTAAGTTCCTCGTGAATCTAGTGAGTTGACGTGTGATCCATCATTTTGTACCCTATGGAGAAAGCCGTAATATACACGAGGTAAGAGTGGCACGAGGTAATTGCGGCACTGGCCAGTAATAAAGAAGACCTAAAAACCCTACTTAA  **3’ end**  TTAAATTGGTGACGAGATTCTTCTTCTATCGTGTGGATTGAGAGGTACATTACCAACCTCATCAACCCTGGTGTCAGGGTTATTATTGAGCCACCAAAGGCCCCTGACATGGCTCATGTAACGACTACTTACTTACATCATTAAGTAGTAACCGGGACCAACGGCTTAACGTGCCTTCCGAAGCACGGATCATCTTACTTTTTGGACAATCAGGTGATCAGCCTGTAATGTCCTAACCAAACTAGGGATCACAAAGTGATTTTTGTGATATGTCCCCACCGGGATTCGAACCCGGGACCTCCGGATCGTGAGCCCAACGCTCAACCACTGGACCACGGAGGTCGTCGTCACGGAGGAGTGACGAGATTAAAGAATAATATACTAACAACGGTAACTCTCCCTCCCCCCGCACCAATTCGTATCGGGCGCCGACCCCCCGGGTCTTACTCTAGTCTTAAATGGTCGTAAGCCGCTAAGGAGTAAGGGGAAGATGTTGTCTCGCTCGCACTTACGGGTTACGCAGCACACGGTAAGAATAAAATTTTCTCATCTACCGACAACTAGGCGTCGGCTAGCATTTCATCCTTATGTTGTGGATATGTCACGTAACTTCAGATTATAATCTATCTCGCGAGACTGTGAACTGTTGAAAAATTGTGAACCGTAACATACTGCTTACAATTTAACATAACGTAACAACATAACAAATACTTTATTGCACAAACAGAAAAAAACAAAATACAAAACAAAAGAGAAATAGAATTAGTACAATAGGCGGCCTTATTGCTAAGTAGCAATCTCTTCCAGGCAACCTTTAAGTATAGGAGATACTGAATAATAAATCGCACCGCCCTCCGCACCACGCCCTTTCTTCGTATCTTTCTAGA |
| OX3402U | Not determined |
