# Supplementary material for: Engineered Repressible Lethality for Controlling the Pink Bollworm, a Lepidopteran Pest of Cotton
Source: PLoS One. 2012 Dec 4;7(12):e50922. doi: 10.1371/journal.pone.0050922 (PMC3514271; doi:10.1371/journal.pone.0050922)
Supplement: Table S2 — Survival to adulthood of transgenic and wild-type progeny of transgene-heterozygous males crossed with wild-type females. Progeny were reared on diet with or without chlortetracycline (CTC and non-CTC, respectively). (DOCX) [file pone.0050922.s002.docx]

|  | **CTC diet** | | **Non-CTC diet** | |
| --- | --- | --- | --- | --- |
| **Strain** | **Wild-type** | **Transgenic** | **Wild-type** | **Transgenic** |
| OX1124A | 20 | 19 | 108 | 36 |
| OX1124C | 193 | 185 | 625 | 166 |
| OX1124D | 104 | 119 | 77 | 22 |
| OX1124E | 22 | 10 | 116 | 53 |
| OX3347A | 558 | 449 | 408 | 0 |
| OX3400A | 485 | 393 | 283 | 22 |
| OX3402A | 25 | 5 | 17 | 0 |
| OX3402C | 77 | 70 | 296 | 0 |
| OX3402M | 37 | 30 | 57 | 0 |
| OX3402P | 46 | 48 | 237 | 0 |
| OX3402T | 49 | 5 | 101 | 0 |
| OX3402U | 60 | 46 | 40 | 0 |
