# Supplementary material for: Engineered Repressible Lethality for Controlling the Pink Bollworm, a Lepidopteran Pest of Cotton
Source: PLoS One. 2012 Dec 4;7(12):e50922. doi: 10.1371/journal.pone.0050922 (PMC3514271; doi:10.1371/journal.pone.0050922)
Supplement: Table S3 — Survival to pupation of transgenic and wild-type progeny of OX3400A-heterozygous males crossed with wild-type females. Progeny were reared on diet with or without chlortetracycline (CTC and non-CTC, respectively), or on host plant material in a field cage. (DOCX) [file pone.0050922.s003.docx]

| **Rearing conditions** | **Wild-type survival** | **OX3400A** |
| --- | --- | --- |
| Lab with CTC | 561 | 495 |
| Lab no CTC | 462 | 90 |
| Field cage | 60 | 0 |
