# Supplementary material for: Engineered Repressible Lethality for Controlling the Pink Bollworm, a Lepidopteran Pest of Cotton
Source: PLoS One. 2012 Dec 4;7(12):e50922. doi: 10.1371/journal.pone.0050922 (PMC3514271; doi:10.1371/journal.pone.0050922)
Supplement: Table S4 — Sequences of primers used for PCR genotyping of strains OX1124A, OX1124D, OX3347A and OX3402A (sequences shown 5′ to 3′). The primers specific for peripheral sequences in the construct were used for all strains. (DOCX) [file pone.0050922.s004.docx]

| **Strain** | **5ʹ flanking sequence primer** | **3ʹ flanking sequence primer** |
| --- | --- | --- |
| OX1124A | GGCAGCATGACCTCTTAATCTTTGC | TCATAGCACCAATAGTCTCGGTGATG |
| OX1124D | GTACCAGCTGATATCCCTCATAACCTACC | CTCCATTAAACATGGTACCCACCAAC |
| OX3347A | AGGTAAGTTGTCTGAAGTTGAG | GAAGGAGTTTTTTACCGCTCCATC |
| OX3402C | TGCACACAGATTAAGTACAGCGTTC | ATGCTAGCTCTTAGAATAGCAAGCCAC |
|  | **Construct 5ʹ terminus** | **Construct 3ʹ terminus** |
| All strains | CTCTGGACGTCATCTTCACTTACGTG | CTCGATATACAGACCGATAAAACACATGC |
