# Supplementary material for: Engineered Repressible Lethality for Controlling the Pink Bollworm, a Lepidopteran Pest of Cotton
Source: PLoS One. 2012 Dec 4;7(12):e50922. doi: 10.1371/journal.pone.0050922 (PMC3514271; doi:10.1371/journal.pone.0050922)
Supplement: Table S5 — Sequences of primers and probes used for real-time PCR of OX3402C samples to quantify tTAV2 expression (sequences shown 5′ to 3′). (DOCX) [file pone.0050922.s005.docx]

| **Target gene** |  | **Name** | **Sequence** |
| --- | --- | --- | --- |
| Endogenous tropomyosin | Primers | PBWtroR | AGATTTCAGGGAGTTACCTACGACCTTT |
|  |  | PBWtroF | CGAGCTCGAGGTCGCCGA |
|  | Probe | PBWtroP | TCGTGTCAAGTCCGGAGACGCCAAG |
| tTAV2 | Primers | tTAVrealF | CCCTGGATATGGCCGATTT |
|  |  | tTAVrealR | gtgttaGCCGCCGTACTCATC |
|  | Probe | tTAVFamP | TCGAGCAGATGTTCACCGACGCC |
